# Evaluating Disease Trends in Marine Ecosystems

**DOI:** 10.1371/journal.pbio.0020119

**Published:** 2004-04-13

After the recent mad cow scare in the United States, 61% of Americans said they would start eating more fish, according to a *Wall Street Journal Online* poll. The respondents may not know that populations of large predatory fish, such as tuna, swordfish, and marlin, have declined 90% over the past 50 years or that less-prized species are increasingly overfished. Or that ever more fish and seafood species show rising levels of mercury contamination, rendering them unfit for human consumption—and contaminating other organisms in the ocean food chain. Humans are also affecting marine life in unexpected ways, as when large numbers of seals in Antarctica in 1955 and in Siberia in 1987 succumbed to canine distemper virus, presumably contracted from domestic dogs. In 2000, more than 10,000 Caspian seals—which also had contact with domestic dogs—died of the same virus. Such human incursions cause even more damage by exacerbating the effects of naturally occurring parasitic and pathogenic diseases that already wreak havoc as they ripple through the food chain.[Fig pbio-0020119-g001]

**Figure pbio-0020119-g001:**
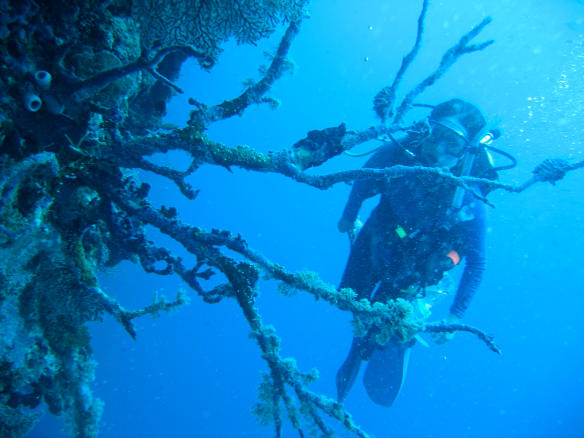
A dead gorgonian sea fan on a wall in Palau (Photograph, with permission, by Drew Harvell)

With recent studies suggesting that disease rates have increased over the past 30 years—and are expected to increase even more, thanks to global climate change—prospects for protecting marine ecosystems depend on understanding the causes and nature of these disease outbreaks. While all indicators point to a real increase in disease rates, scientists have no baseline data to measure these increases against and so cannot directly test the hypothesis that marine diseases are increasing. Now Jessica Ward and Kevin Lafferty report a method that uses the recorded incidence of disease as a proxy for baseline data to identify disease trends in major groups of marine organisms.

Ward and Lafferty conducted an online search of 5,900 journals published from 1970 to 2001 for reports of disease in nine taxonomic groups: turtles, corals, mammals, urchins, mollusks, seagrasses, decapods (crustaceans), sharks/rays, and fishes. Their approach takes into account three potentially confounding factors in determining trends in this type of search. Fluctuations in publication numbers could skew results, since an increase in the number of scientific reports published in a particular taxonomy might not reflect a true increase in the incidence of disease; a particularly prolific author could bias the search results by turning up more cases of disease in a population than actually occurred; or a single disease event reported multiple times in different papers could create the impression that disease had suddenly increased. To normalize publication rates over time, Ward and Lafferty used a proportion of disease reports from a given population relative to the total number of reports in that group. To determine whether there was an “author effect,” they removed the most prolific author in each taxonomic group and found that an author's abundant contributions did not skew the results. Finally, they confirmed that a single disease didn't bias their results by removing multiple reports of the same disease from the literature before analyzing the trends.

When they analyzed the searches without adjusting for the total number of reports published, Ward and Lafferty found that reports of disease increased for all groups. But when they analyzed the normalized results, they found that trends varied. While there was a clear increase in disease among turtles, corals, mammals, urchins, and mollusks, they found no significant trends for seagrasses, decapods, and sharks/rays. And they found that disease reports actually decreased for fishes. (One explanation for this decrease could be that drastic reductions in population density present fewer opportunities for transmitting infection.) Ward and Lafferty tested the soundness of this approach by using a disease (raccoon rabies) for which baseline data exist and showing that normalized reports of raccoon rabies increased since 1970, just as the disease increased from one case reported in Virginia in 1977 to an “epizootic” outbreak, affecting eight mid-Atlantic states and Washington, D.C., by 1992.

The pattern of increased reports, the authors propose, confirms scientists' perceptions about the rising distress of threatened populations and thus reflects a real underlying pattern in nature. The fact that disease did not increase in all taxonomic groups suggests that increases in disease are not simply the result of increased study and that certain stressors, such as global climate change, likely impact disease in complex ways. By demonstrating that an actual change in disease over time is accompanied by a corresponding change in published reports by scientists, Ward and Lafferty have created a powerful tool to help evaluate trends in disease in the absence of baseline data. It is only by understanding the dynamics of disease outbreaks that scientists can help develop sound methods to contain them.

